# Conservation of folding and association within a family of spidroin N-terminal domains

**DOI:** 10.1038/s41598-017-16881-6

**Published:** 2017-12-01

**Authors:** Julia C. Heiby, Suhaila Rajab, Charlotte Rat, Christopher M. Johnson, Hannes Neuweiler

**Affiliations:** 1 Department of Biotechnology & Biophysics, Julius-Maximilians-University Würzburg, Am Hubland, 97074 Würzburg, Germany; 2 Medical Research Council Laboratory of Molecular Biology, Francis Crick Avenue, Cambridge, CB2 0QH United Kingdom

## Abstract

Web spiders synthesize silk fibres, nature’s toughest biomaterial, through the controlled assembly of fibroin proteins, so-called spidroins. The highly conserved spidroin N-terminal domain (NTD) is a pH-driven self-assembly device that connects spidroins to super-molecules in fibres. The degree to which forces of self-assembly is conserved across spider glands and species is currently unknown because quantitative measures are missing. Here, we report the comparative investigation of spidroin NTDs originating from the major ampullate glands of the spider species *Euprosthenops australis*, *Nephila clavipes*, *Latrodectus hesperus*, and *Latrodectus geometricus*. We characterized equilibrium thermodynamics and kinetics of folding and self-association using dynamic light scattering, stopped-flow fluorescence and circular dichroism spectroscopy in combination with thermal and chemical denaturation experiments. We found cooperative two-state folding on a sub-millisecond time scale through a late transition state of all four domains. Stability was compromised by repulsive electrostatic forces originating from clustering of point charges on the NTD surface required for function. pH-driven dimerization proceeded with characteristic fast kinetics yielding high affinities. Results showed that energetics and kinetics of NTD self-assembly are highly conserved across spider species despite the different silk mechanical properties and web geometries they produce.

## Introduction

The question of how a linear chain of amino acids spontaneously folds into a highly ordered three-dimensional structure continues to be a central topic in molecular biology^1^. Important insights come from comparative studies of homologous proteins, where differences in folding mechanisms can be traced back to minor sequence changes^2–4^. Such studies allow the dissection of the roles of sequence and topology in folding. A class of highly evolved proteins that exhibit rather unusual amino acid composition are the silk proteins produced by web spiders. Spider silk is produced by the controlled assembly of spider fibroins, so-called spidroins, within the spinning gland of the animal yielding threads of outstanding mechanical properties tailored for distinct functionalities. Material scientists are trying to decrypt the assembly process and to reproduce it in the laboratory^5–7^. The bulk of a spidroin sequence consists of repetitive poly-alanine and glycine-rich peptide motifs of simple amino acid composition, which are unstructured under storage conditions in the gland and form mainly β-sheet secondary structure in solid fibres. The repetitive central segments are terminated by the globular folded N- and C-terminal domains (NTD and CTD), which provide water-solubility on the one hand and connectivity in response to mechanical and chemical stimuli on the other^8,9^. NTD and CTD are five-helix bundles that form homo-dimers. While the CTD is a covalent homo-dimer stabilized by a disulfide linkage, the NTD is monomeric under storage conditions in the gland and undergoes self-association in the spider’s spinning duct in response to changes of solution pH and salt composition, thus polymerizing spidroins to form super-molecules^7,10^. pH-triggered NTD self-association is ultrafast and involves site-specific protonation events and conformational change^11–15^.

NTD and CTD represent the most conserved sequence areas of spidroins, with no structural homologues identified so far, underscoring their importance in the process of silk formation^8,10^. Interestingly, the unusual amino acid composition of the central spidroin segments extends into the terminal domains: in the NTD, alanine is the most frequently found amino acid followed by serine; alanine and serine together take up ~30% of an NTD sequence. By contrast, the number of charged side chains is rather low. This is surprising considering the high water-solubility of the domain, which is commonly provided by side chain charges.

Unusual amino acid composition and high degree of sequence conservation make spidroin NTDs an interesting system both from the viewpoint of fundamental folding research and material science. Open questions are: does the unusual amino acid composition of NTDs translate into an unusual mechanism of folding? Are energetics and kinetics of folding and self-association conserved? This question is in particular interesting in light of species-dependent differences in strengths and structures of silks^16^.

Here, we report the comparative investigation of folding and association of NTDs originating from major ampullate spidroin 1 (MaSp1) of four different spider species, namely the black widow (*Latrodectus hesperus*, Lh) the brown widow (*Latrodectus geometricus*, Lg), the golden orb spider (*Nephila clavipes*, Nc), and the nursery web spider (*Euprosthenops australis*, Ea). The Ma gland forms the toughest fiber used to build the web frame or a lifeline and is a focus of current material science. We found that all four domains folded on a similar sub-millisecond time scale via a conventional two-state mechanism. The Nc homologue, however, was significantly less stable and folded more slowly compared with the other domains. Rate constants of pH-triggered domain self-association and dissociation were similar. Results showed that, despite species-dependent differences in web geometries and silk mechanical properties, the energetics and kinetics of NTD self-assembly are conserved.

## Results

### Self-association of spidroin NTDs

**Figure 1:**
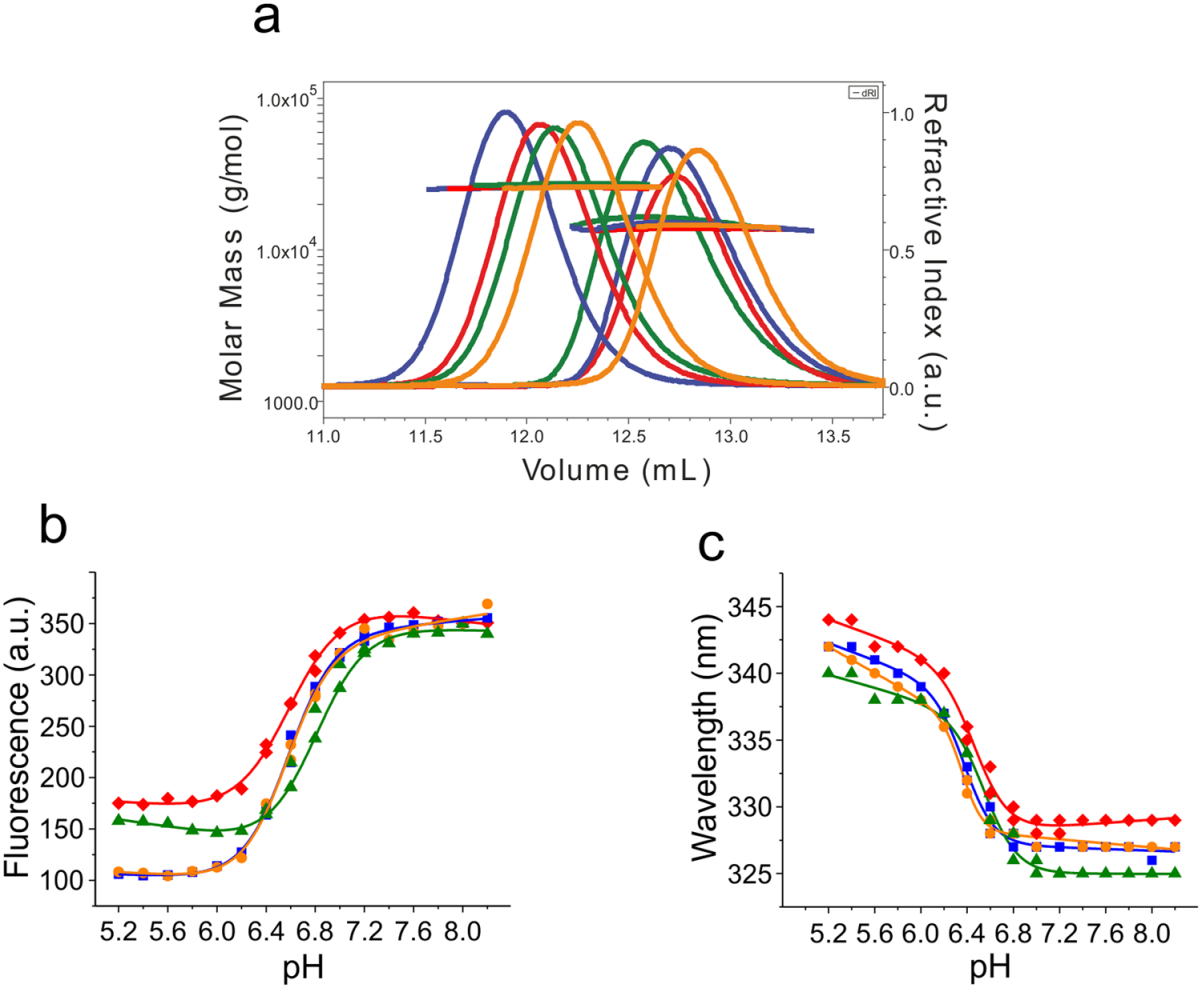
Functionality of spidroin NTDs. (**a**) SEC-MALS chromatograms of NTDs under monomer and dimer solution conditions showing refractive index against elution volume. The evaluated mass from light scattering analysis is indicated by the horizontal lines. Lh (blue), Lg (orange), Nc (green), and Ea (red). (**b**) Steady-state tryptophan fluorescence emission intensities of Lh (blue squares), Lg (orange circles), Nc (green triangles), and Ea (red dimonds) NTD recorded under conditions of varying solution pH. Trp fluorescence emission was recorded at 330 nm and normalized to the maximal emission intensity for reasons of clarity. (**c**) Wavelength of maximal fluorescence emission of the four homologues recorded under conditions of varying solution pH (color code from above applies). Solid lines are guides to the eye.

We produced NTDs of spidroin 1 from the major ampullate gland (MaSp1) of the species Ea, Lh, Lg, and Nc using heterologous overexpression in *E. coli* bacterial cells followed by chromatographic purification. We characterized self-association using analytical size-exclusion chromatography in combination with multi-angle light scattering (MALS) spectroscopy (SEC-MALS). NTDs were investigated under conditions that favor either the monomeric state, i.e. in 50 mM aqueous phosphate buffer pH 7.0 with the solution ionic strength (*I*) adjusted to 200 mM using potassium chloride, or the dimeric state, i.e. in 20 mM (N-morpholino)ethansulfonic acid (MES) pH 6.0 with the *I* adjusted to 60 mM. Molecular mass moments of SEC-MALS experiments confirmed that the domains were either monomers or dimers under these conditions and that they were able to undergo functional dimerization (Fig. 1a).

MaSp1 NTDs contain a single tryptophan (Trp) that changes conformation from buried to solvent-exposed upon dimerization of the native protein^8,13–15^. Trp is more solvent exposed in the dimer so the fluorescence of this state is lower. We recorded Trp fluorescence emission intensities of the four homologues under solution conditions of varying pH (pH between 5 and 8). We observed strong fluorescence quenching and bathochromic shifts of fluorescence emission maxima at low pH, in agreement with increased solvent exposure of the Trp side chain upon dimerization (Fig. 1b,c). Minor differences in fluorescence amplitudes and wavelengths of maximal emission intensity measured between low and high pH may be explained by minor differences in tertiary packing and degrees of solvent-exposure of the Trp side chains of the different homologues.

**Figure 2:**
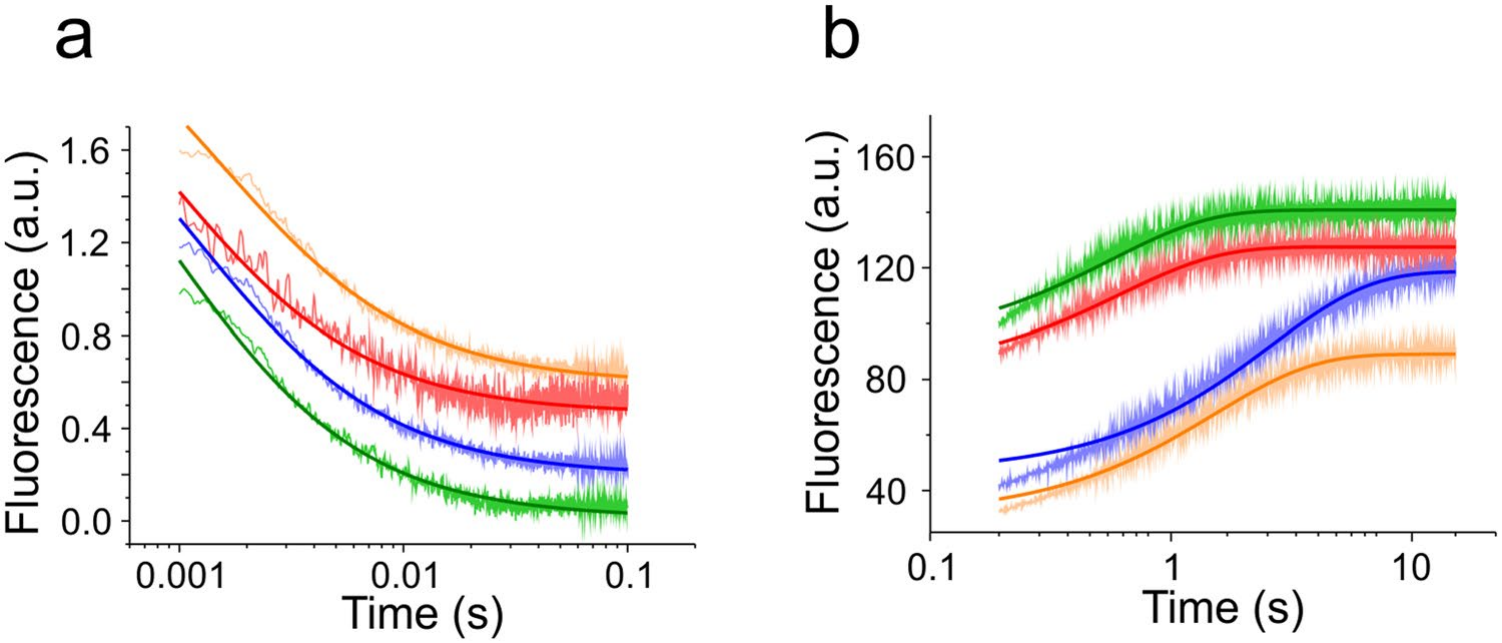
Kinetics of association and dissociation. (**a**) Representative transients of association recorded using stopped-flow Trp fluorescence spectroscopy (Lh: blue, Lg: orange, Nc: green, Ea: red). 500 nM NTD was rapidly mixed from pH 7.0 into pH 6.0 buffered solution. Each trace is an average over 24 single shots. The traces are offset by 0.2 a.u. along the y-axis for reasons of clarity. Dark coloured lines represent data fits using a kinetic model of dimerization. (**b**) Representative transients of NTD dissociation measured by stopped-flow AttoOxa11 fluorescence spectroscopy (Lh: blue, Lg: orange, Nc: green, Ea: red). Transients were recorded by chasing 100 nM fluorescently modified NTD prepared at pH 6.0 with tenfold excess of non-labelled NTD prepared at same solution conditions. Traces are offset along the y-axis for reasons of clarity. Dark coloured lines are mono-exponential data fits.

We measured kinetics of NTD self-association using rapid mixing experiments. A stopped-flow apparatus was used to rapidly change solution pH and *I* from monomer to dimer conditions. Kinetics of self-association was followed by decaying Trp fluorescence intensities, which signalled dimer formation (Fig. 2a). We fitted a kinetic model of dimerization to the measured fluorescence intensity transients in order to obtain the microscopic rate constants of self-association, *k*_ass_. Association of each homologue was similarly fast, i.e. *k*_ass_ was on the order of 10^9^ M^−1^ s^−1^ (Table 1). Rate constants of dissociation, *k*_diss_, were measured using chasing experiments. NTD dimerization can be detected by fluorescence self-quenching of an extrinsic label at sequence position 50, which is located at the rim of the association interface^11^. We labelled the oxazine fluorophore AttoOxa11 to sequence position 50 of all four homologues using thiol modification of single-point cysteine (Cys) mutants. One labelled subunit of fluorescence self-quenched homo-dimers prepared in pH 6 buffer was chased off by rapidly mixing in an excess of non-labelled protein. Dissociation was thus signalled by an increase of fluorescence emission intensity of the AttoOxa11 label. The fluorescence transients obtained from stopped-flow experiments were fitted using an exponential function, yielding *k*_diss_ (Fig. 2b). Minor deviations from the exponential fit at fast time scale are likely explained by the presence of small populations of oligomeric species formed under solution conditions that favour fibre formation. Rate constants of dissociation of the four homologues were similar, i.e. *k*_diss_ was on the order of 1 s^−1^ (Table 1). Using these kinetic results we calculated the equilibrium dissociation constants of dimers, *K*_d_ = *k*_diss_/*k*_ass_, which were all in the low nanomolar range (Table 1).

**Table 1:** Rate constants of association and dissociation of NTDs.

Construct	*k*_ass_	*k*_diss_	*K*_d_
	(10^9^ M^−1^·s^−1^)	(s^−1^)	(10^−9^ M)
*L. hesperus*	2.19 ± 0.30	0.31 ± 0.02	0.14 ± 0.01
*L. geometricus*	1.39 ± 0.12	0.64 ± 0.02	0.46 ± 0.03
*N. clavipes*	2.58 ± 0.20	1.88 ± 0.05	0.73 ± 0.04
*E. australis*	1.58 ± 0.24	1.68 ± 0.43	1.07 ± 0.11

### Equilibrium denaturation of NTD monomers

**Figure 3:**
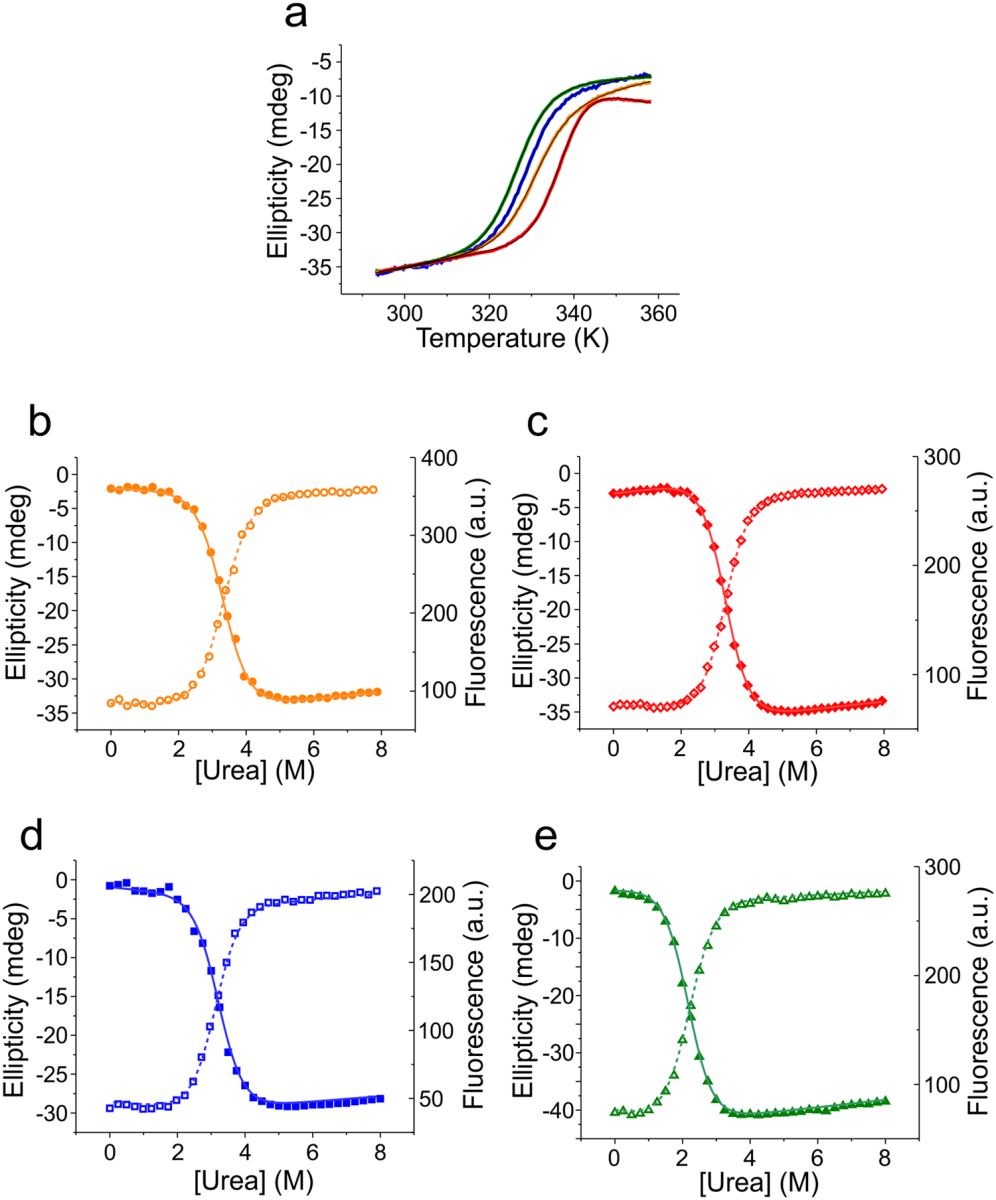
Equilibrium denaturation of NTD monomers. (**a**) Normalized melting curves of NTDs recorded at pH 7.0 abbreviation for ionic strength: *I* adjusted to 200 mM using far-UV CD spectroscopy at 222 nm (Ea: red, Lh: blue, Lg: orange, Nc: green). Black lines are data fits using the thermodynamic model for a two-state equilibrium. (**b**–**e**) Equilibrium chemical denaturation. Data were recorded by manual titration using urea as denaturant in pH 7.0 buffered solution with *I* adjusted to 200 mM ((**b**) Lg, orange circles; (**c**) Ea, red diamonds; (**d**) Lh, blue squares; (**e**) Nc, green triangles). Denaturation was measured using far-UV CD spectroscopy at 222 nm (closed symbols) and Trp fluorescence spectroscopy at 330 nm (open symbols). Solid lines are data fits using the thermodynamic model for a two-state equilibrium.

To investigate the folding equilibrium of NTDs we performed thermal and chemical denaturation experiments using circular dichroism (CD) and Trp fluorescence spectroscopy. In order to avoid complications in data analysis that would arise from an additional monomer/dimer equilibrium, denaturation was performed in pH 7 buffered solutions where *I* was adjusted to 200 mM - conditions where the NTDs were monomeric. The CD signal at 222 nm was used as a probe for α-helical secondary structure. Tertiary structure was probed by native Trp fluorescence emission. Thermal denaturation data acquired using CD spectroscopy were well described by a thermodynamic two-state model of folding (Fig. 3a). Fitted enthalpies and melting temperatures are shown in Table 2. Similarity of enthalpies of unfolding indicated similarly strong, non-covalent intramolecular interaction networks that stabilize the fold. The slightly higher enthalpy obtained for Ea may be explained by the observed irreversibility of thermal unfolding of this homologue.

**Table 2:** Thermal denaturation data.

NTD homologue	*L. hesperus*	*L. geometricus*	*N. clavipes*	*E. australis*
T_m_ (K)	329.9 ± 0.1	331.0 ± 0.1	326.8 ± 0.1	337.0 ± 0.1
ΔH_m_ (kcal/mol)	54.8 ± 0.7	50.1 ± 0.4	54.3 ± 0.2	67.5 ± 0.4

Chemical denaturation was performed using urea as denaturant and monitored using CD and Trp fluorescence spectroscopy. The denaturation transitions obtained by both methods were well described by a two-state model invoking a linear free energy relationship (Fig. 3b–e). For each homologue, the equilibrium *m*-values and transition mid-points from CD and Trp fluorescence data were in good agreement (Table 3). Free energies of unfolding were similar, except for the Nc homologue, which had a significantly reduced stability that arose from a lower denaturation mid-point (Table 3).

**Table 3:** Equilibrium chemical denaturation data.

NTD homologue	Signal	*m*_D-N_	[Urea]_50_%	Δ*G*_D-N_
		(kcal·M^−1^·mol^−1^)	(M)	(kcal·mol^−1^)
*L. hesperus*	CD	1.64 ± 0.04	3.09 ± 0.01	5.07** ± **0.14
	Fluo	1.58 ± 0.08	3.20 ± 0.02	5.06** ± **0.29
*L. geometricus*	CD	1.46 ± 0.05	3.39 ± 0.02	4.95** ± **0.20
	Fluo	1.53 ± 0.06	3.34 ± 0.02	5.10** ± **0.23
*N. clavipes*	CD	1.59 ± 0.04	2.19 ± 0.02	3.48** ± **0.12
	Fluo	1.67 ± 0.04	2.18 ± 0.01	3.64** ± **0.10
E. australis*	CD	1.69 ± 0.03	3.30 ± 0.01	5.60** ± **0.12
	Fluo	1.69 ± 0.02	3.31 ± 0.01	5.60** ± **0.08

*Data from ref.^23^.

### Folding kinetics of NTD monomers

**Figure 4:**
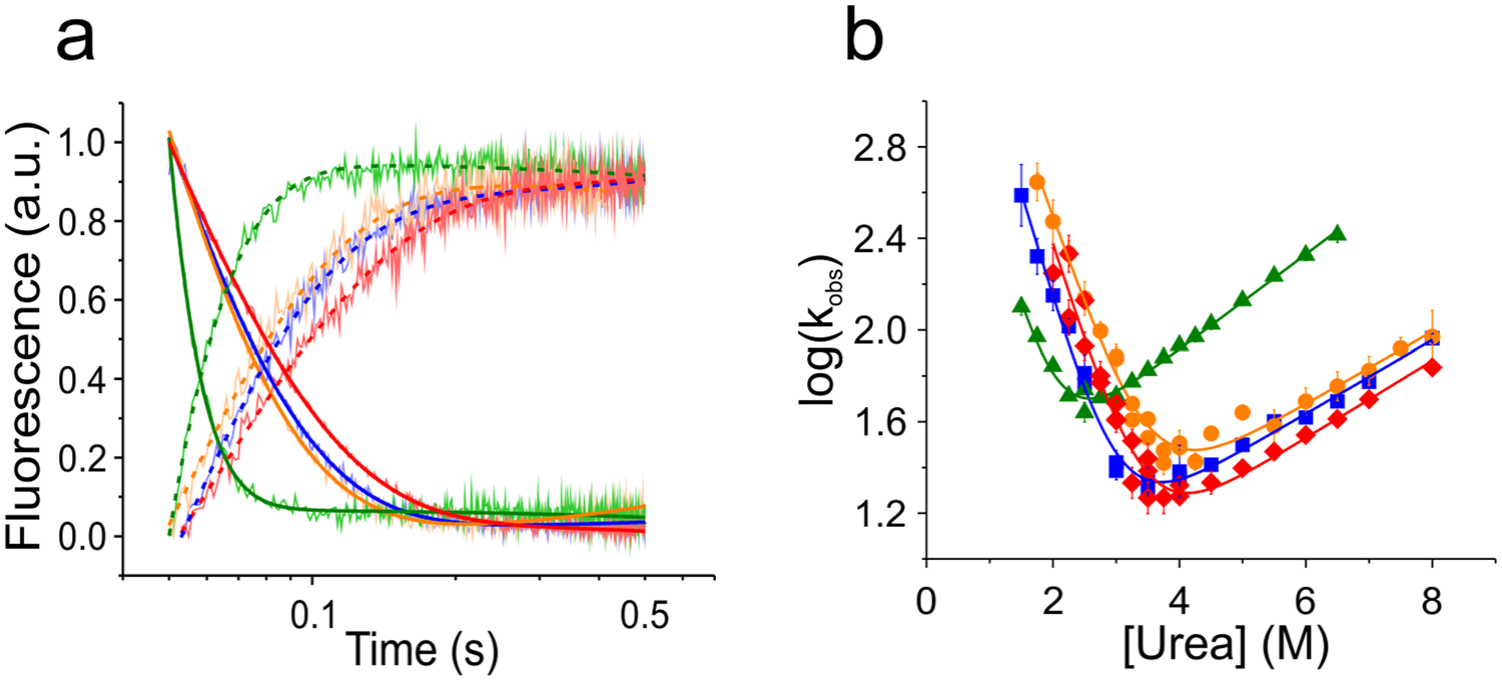
Folding kinetics. (**a**) Representative kinetic transients of folding (decay) and unfolding (rise) measured using stopped-flow Trp fluorescence spectroscopy (Lh: blue, Lg: orange, Nc: green, Ea: red). Each kinetic trace shown in light colour is a single shot. Solid lines and dashed lines are mono-exponential fits to unfolding and folding data, respectively. Data are normalized to 1 for reasons of clarity. Shown unfolding traces were measured at 5 M urea and shown folding traces were measured at 3 M urea. (**b**) Observed relaxation rate constants plotted versus urea concentration (chevron analysis). Solid lines are fits to the data using a kinetic model for a barrier-limited two-state transition. Same colour code as in (**a**) applies.

We measured kinetics of folding using chemical denaturation and stopped-flow Trp fluorescence spectroscopy. NTD samples were prepared in pH 7 buffer at either zero or six molar urea and were rapidly mixed to higher or lower denaturant concentrations to either unfold or refold the protein. Obtained Trp fluorescence intensity time traces were fitted using single-exponential functions containing a linear baseline drift (Fig. 4a). The linear baseline drift occurred on a slow time scale and can be explained by photo-bleaching or sample diffusion in the mixing zone of the stopped-flow machine. For each homologue we plotted the observed rate constants *versus* denaturant concentration and performed chevron analysis (Fig. 4b). Observed rate constants fitted well to a kinetic model for a barrier-limited two-state transition. The extrapolated microscopic rate constants of folding and unfolding of the homologues Ea, Lh, and Lg ranged between *k*_f_ = 10,000–13,000 s^−1^ and *k*_u_ = 3–5 s^−1^ (Table 4). The Nc homologue had a slower rate constant of folding and a faster rate constant of unfolding compared with its family members, in agreement with its lower stability found in equilibrium experiments (Tables 3 and 4). Thermodynamic quantities derived from kinetics were in good agreement with values derived from equilibrium data. The sum of kinetic folding and unfolding *m*-values (*m*_f_ + *m*_u_) compared well with the respective equilibrium *m*-values, *m*_eq_. Equilibrium free energies were in reasonable agreement with quantities calculated from *k*_f_ and *k*_u_ (Δ*G* = −RT ln(*k*_u_/*k*_f_)), supporting a two-state model of folding model^17^. Tanford β-values (β_T_ = *m*_f_/(*m*_f_ + *m*_u_)) were all around 0.85 (Table 4) showing that folding proceeded through a similarly compact, native-like transition state.

**Table 4:** Kinetics of folding.

Homologue	*k*_f_	*m*_f_	*k*_u_	*m*_u_	Δ*G*_D-N_	β_T_
*L. hesperus*	10 123 ± 2 278	1.29 ± 0.06	4.67 ± 0.70	0.22 ± 0.01	4.55** ± **0.16	0.85 ± 0.01
*L. geometricus*	11 899 ± 3 035	1.09 ± 0.06	5.34 ± 1.16	0.22 ± 0.02	4.56** ± **0.20	0.83 ± 0.01
*N. clavipes*	3 652 ± 1 328	1.39 ± 0.13	12.16 ± 1.02	0.28 ± 0.01	3.38 ± 0.22	0.83 ± 0.01
*E. australis*	13 257 ± 6 348	1.20 ± 0.11	3.28 ± 1.14	0.23 ± 0.03	4.92** ± **0.35	0.84 ± 0.02

*k*_f_ and *k*_u_ (s^−1^); *m*_f_ and *m*_u_ (kcal·M^−1^·mol^−1^); Δ*G*_D-N_ (kcal/mol).

### Influence of solution ionic strength on folding of NTD monomers

**Figure 5:**
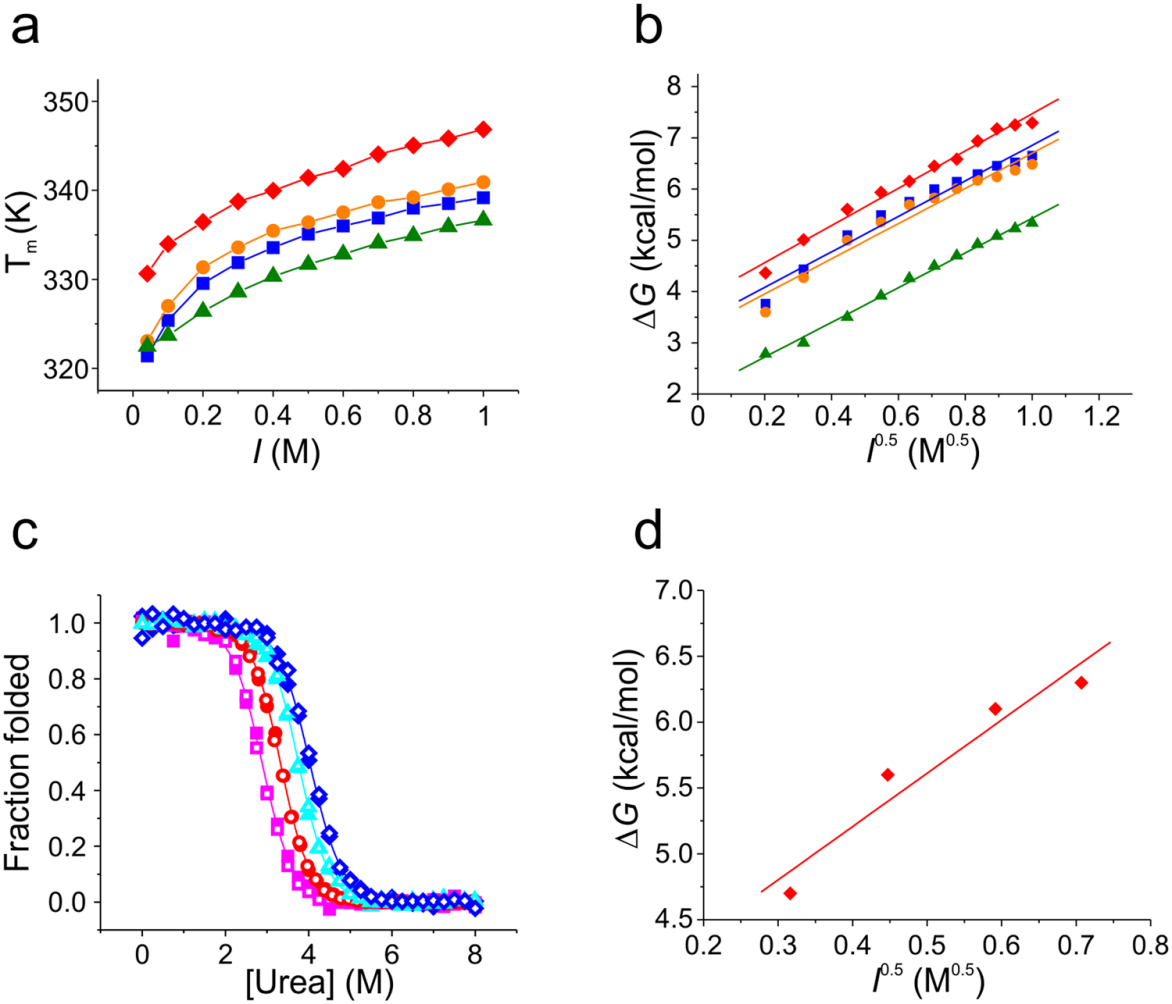
Influence of salt on NTD monomer folding and stability. (**a**) Dependence of thermal stability on *I* measured in pH 7.0 buffered solutions using far-UV CD spectroscopy at 222 nm. Thermal denaturation mid-points are plotted versus *I* (Lh: blue squares, Lg: orange circles, Nc: green triangles, Ea: red diamonds). Solid lines are a guide to the eye. (**b**) Changes of free energy plotted versus the square root of *I*. Solid lines are linear data fits. Same colour code as in (a) applies. (**c**) Chemical denaturation of the Ea NTD measured in pH 7.0 buffered solution using far-UV CD (closed symbols) and Trp fluorescence spectroscopy (open symbols). Data were normalized to the folded fraction. Solid lines are data fits using a thermodynamic model for a two-state equilibrium. Magenta squares: 100 mM *I*; red circles: 200 mM *I*; cyan triangles: 350 mM *I*; blue diamonds: 500 mM *I*. (**d**) Plot of the change of free energy versus the square root of *I* measured using chemical denaturation. The solid line is a linear fit to the data.

To investigate the electrostatic contribution to stability, we studied the influence of *I* on the folding of NTDs. We recorded thermal denaturation data of each homologue using CD spectroscopy in pH 7 buffered solutions with *I* ranging between 0.041 M and 1.00 M. Results showed that the stability of all four homologues increased similarly with increasing *I*, which was evident from the observed increase of thermal denaturation midpoints (Fig. 5a). Findings were in agreement with previous observations made for the Lh homologue^15^. We found that the observed increase of stability was the result of Debye-Hückel screening of point charges^18^: plots of Δ*G* versus the square root of *I* (*I* ^0.5^) were linear for each protein (Fig. 5b). The slope of a linear fit to Δ*G* plotted versus *I* ^0.5^ can be interpreted as an equilibrium *m*-value that accounts for the effect if *I* on stability, *m*_eq_’^19^, in analogy to the well-known *m*_eq_ in the classical linear free-energy relationship that applies to denaturants. From this analysis we found that the electrostatic contribution to stability was highly conserved within the NTD family, i.e. *m*_eq_’ of Ea, Nc, Lh, and Lg was 3.6 ± 0.2, 3.4 ± 0.1, 3.5 ± 0.2, and 3.4 ± 0.3 kcal mol^−1^ M^−0.5^, respectively. The result was confirmed by chemical denaturation experiments performed under conditions of varying *I* on the Ea homologue. Denaturation data showed mid-points that increased with *I* while the classical equilibrium *m*-value, *m*_eq_, remained unchanged (Fig. 5c). A linear fit to the plot of Δ*G* versus *I* ^0.5^ yielded *m*_eq_’ = 4.0 ± 0.8 kcal mol^−1^ M^−0.5^ (Fig. 5d), a value that was within error of the one obtained from thermal denaturation data.

## Discussion

Web spiders use up to seven specialized glands to synthesize silk fibres for various tasks including prey capture, reproduction and shelter^6,7^. The basic principles of synthesis are thought to be conserved across glands and species. Conservation of mechanism is reflected in the conserved, modular sequence architecture of spidroins, which are the building blocks of silk. Yet, sizes, geometries, and mechanical properties of webs built by different spider species vary strongly. These differences may arise from sequence variations in the central, repetitive spidroin segments that make up the bulk of interactions in silk and possibly from different processing conditions in various spinning ducts^5,6,16^. Little is known about the contribution of sequence modulations in the terminal domains to modulation of silk. Structural studies show that MaSp NTD folds from Ea, Lh, and Nc, as well as from a homologue of the minor ampullate gland of *Araneus ventricosus* ^8,15,20,21^ are conserved, although minor differences in helix packing are observed^20^. Biophysical measurements show similar signatures of pH- and salt-dependent dimerization of the *Euprosthenops*, *Latrodectus*, *Nephila* and *Araneus* NTDs^13–15,21^. However, quantitative measures of folding and self-association were missing and are reported here.

Our comparative study of MaSp1 NTDs from Ea, Nc, Lh, and Lg shows similar intensity-loss and red-shift of Trp fluorescence emission upon dimerization (Fig. 1b). Stopped-flow fluorescence experiments showed that the high speed of pH-triggered self-association was similar in all four homologues and responsible for tight binding, with *K*_d_ values in the low nM range (Table 1). Structural studies report subtle rearrangement of helices and a consequently altered dimer interface of the Nc homologue^20^. We found that these structural rearrangements did apparently not translate into a modulation of dimerization kinetics or strength of association. Our results suggest that the MaSp1 NTD evolved as a pH-driven module that connects spidroins in the distal part of the gland by same mechanism and energetics irrespective of species. Findings underscore the importance of species-dependent sequence modulations in the central, repetitive segments for modulation of silk mechanical properties.

The folding of all four homologues appeared cooperative and was well described by two-state transitions - a behaviour that is frequently observed for small, single-domain proteins^22^. Cooperativity of folding was evident from the good agreement of thermodynamic quantities measured using two different structural probes and from the overall good agreement of quantities derived from equilibrium and kinetic experiments. We found remarkably fast sub-millisecond kinetics of folding of all four homologues (Table 4). Chevron analysis of folding kinetics supported the two-state model with no indications of populated folding intermediates. The kinetic *m*-value and extrapolated rate constant of folding of the Ea NTD was within error of the values previously estimated from temperature-jump experiments^23^. Fast kinetics of folding suggest MaSp1 NTDs as an interesting family for future combined experimental and computational studies that can access overlapping time scales^24^. Such studies yield atomic-detailed insights into pathways of folding^25^.

**Figure 6:**
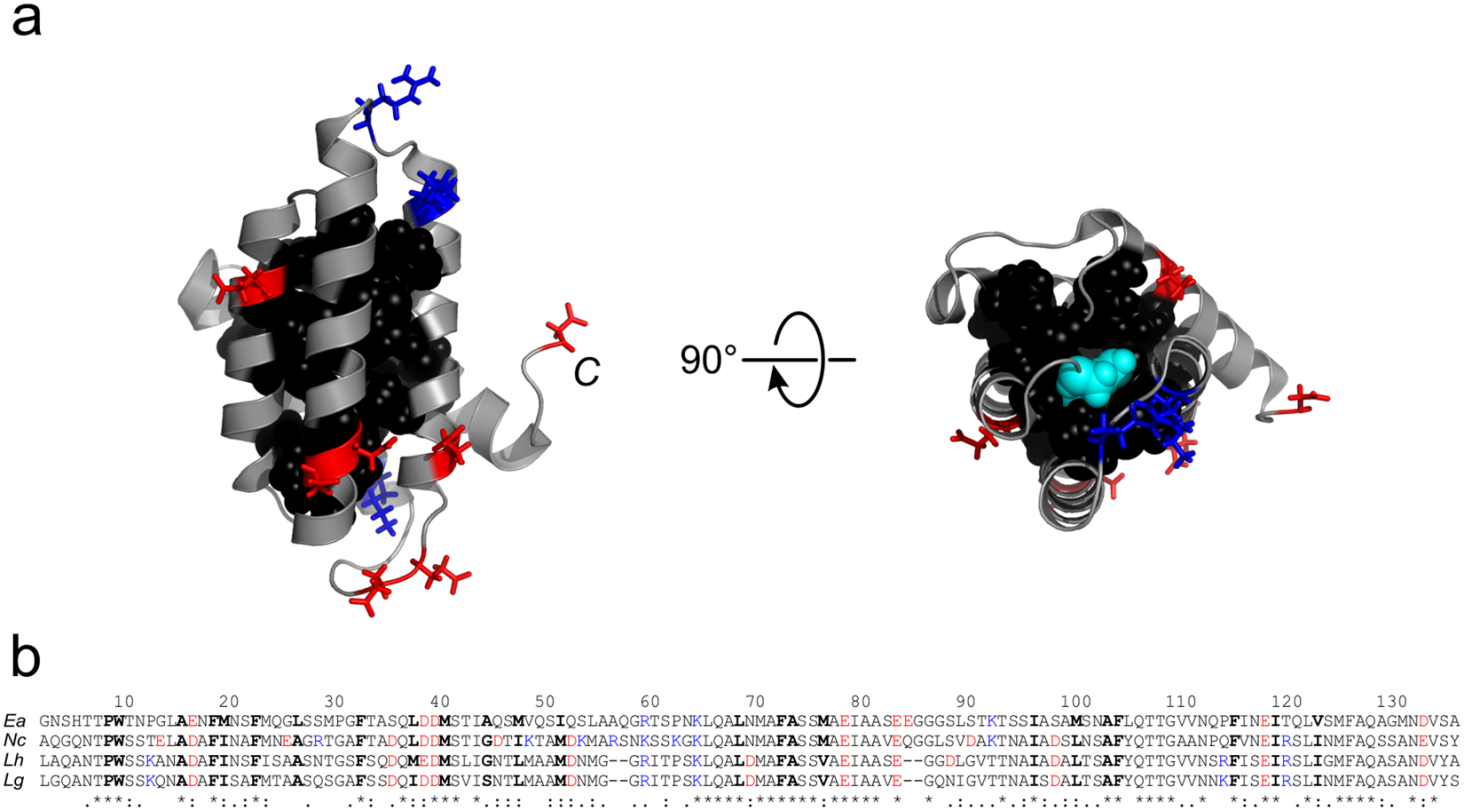
Structure and sequence alignment of MaSp1 NTDs. (**a**) Solution structure of the monomeric Ea NTD (pdb id 2LPJ). Amino acid side chains that form the hydrophobic core are shown as van-der-Waals spheres (black). Acidic and basic side chains are highlighted in red and blue stick representation. The C-terminus of the domain is indicated. The panel on the right hand side shows a top view on the domain highlighting the conserved N-terminal Trp buried in core position (W10, cyan spheres). (**b**) Sequence alignment of Ea, Nc, Lh, and Lg NTD (from top to bottom). Acidic and basic side chains are highlighted red and blue. Side chains that form the hydrophobic core are highlighted black and bold. Identical (*), very similar (:) and similar (.) side chains are indicated at the bottom.

The fold of spidroin NTDs is stabilized by an extensive hydrophobic core (Fig. 6a). Observed similarity of stabilities may be explained by the high degree of conservation of residue side chains that form the core: 15 of 24 core side chains are identical and the remaining 9 are of high similarity (Fig. 6b). This raises the question as to the origin of reduced stability of the Nc homologue compared with its family members (Tables 3 and 4). The subtle rearrangement of helices found in structural studies^20^ and the resulting change of the tertiary interaction network may explain the finding.

The number of ionisable side chains in NTDs is low compared with the average content of 29% found in proteins^26^ (Fig. 6). The few side chain charges, however, fulfil critical roles in the mechanism of pH-triggered self-association. Basic and acidic residue side chains cluster on opposing poles of the domain generating a macromolecular dipole that steers anti-parallel orientation in the dimeric assembly^8^. Some specific acidic side chains are involved in the pH-relay mechanism that locks the dimer^8,11,12,20^. Clustering of point charges, however, compromises stability of the domains through intramolecular, repulsive electrostatic forces. There is thus a trade-off of stability of the monomeric fold versus stability of the dimeric assembly, both modulated by electrostatics. Whilst high concentrations of salt stabilize the monomeric fold, they destabilizes the dimeric assembly. Decreasing concentrations of sodium chloride found along the spinning duct towards the tapering end where the fibre is formed^27^ thus stabilizes the dimer. Salt ions in solution can alleviate intramolecular electrostatic strain either through direct binding or by the effect of Debye-Hückel screening. The latter is explained by the formation of clouds of counter-ions around point charges that screen Coulombic forces. This screening is related to the thickness of the ion cloud, which depends on the square root of *I* ^18,19^. We found a linear dependence of the free energy of folding on the square root of *I* for all NTDs and thus identified Debye-Hückel screening as the mechanism of salt action on this domain. Although the Nc homologue exhibits the highest number of side chain charges (Fig. 6b) additional electrostatic strain appears not to be the origin of the reduced stability compared with the other homologues: *m*_eq_’ of all four domains was similar. The mean *m*_eq_’ of 3.5 ± 0.1 kcal mol^−1^ M^−0.5^ was higher than the value reported for the small protein FynSH3 (*m*_eq_’ = 2.90 ± 0.07 kcal mol^−1^ M^−0.5^)^19^. The higher *m*_eq_’ of NTDs likely arises from the highly localized clustering of point charges that steers anti-parallel orientation during dimerization at the expense of a reduced stability of the fold.

In conclusion, our comparative study of folding and association within a family of MaSp1 NTDs reveals kinetics and energetics of self-assembly that are conserved across species. Small size, unusual sequence properties and high speed of folding and binding suggests the NTD family as an interesting system for future combined experimental and computational studies that may elucidate mechanisms of conformational change and self-assembly at atomic detail. Moreover, it will be interesting to see if conservation of folding and association also holds for spidroin NTDs originating from glands other than those used to spin dragline silk.

## Methods

### Protein synthesis, mutagenesis and fluorescence modification

Synthetic genes of NTDs from MaSp1 of the spider species Lh, Lg, Nc and Ea (GeneArt, Thermo Fisher Scientific) were cloned into a modified pRSETA vector (Invitrogen, Thermo Fisher Scientific) using conventional restriction digestion and ligation protocols. The gene constructs contained an N-terminal His_6_-tag followed by a thrombin recognition sequence for proteolytic removal of the tag. Single-point cysteine mutants (Q50C) were generated using the QuikChange mutagenesis protocol (Stratagene). Recombinant NTDs were overexpressed in *Escherichia coli* C41 (DE3) bacterial cells. NTDs and mutants thereof were isolated from clarified cell lysate by affinity chromatography using Ni-Sepharose 6 Fast-Flow resin (GE Healthcare) followed by proteolytic cleavage of the His_6_-tag using thrombin from bovine plasma (Sigma-Aldrich). Proteins were purified to homogeneity using size exclusion chromatography (SEC) on a Superdex 75 column (GE Healthcare) equilibrated with 200 mM ammonium bicarbonate. Pooled protein fractions were lyophilized. Purity of protein samples was confirmed by SDS-PAGE. Q50C mutants were modified with the thiol-reactive maleimide derivative of the oxazine fluorophore AttoOxa11 (Atto-Tec). Labelling was carried out using a 5-fold molar excess of fluorophore reacted for 2.5 h at 298 K in 50 mM 3-(*N*-morpholino)propanesulfonic acid (MOPS), pH 7.5, containing 6 M guanidinium chloride and a 10-fold molar excess of tris(2-carboxyethyl)phosphine (TCEP) to prevent thiol oxidation. Labelled protein was isolated from non-reacted dye using SEC (Sephadex G-25 resin, GE Healthcare).

### SEC-MALS experiments

NTD samples for SEC-MALS (100 µL at 5 mg/mL) were resolved on a Superdex-75 HR10/300 analytical gel filtration column (GE Healthcare) at 0.5 ml min^−1^ in either 50 mM phosphate, pH 7.0, with *I* adjusted to 200 mM using KCl (monomer conditions) or 20 mM MES, pH 6.0, with *I* adjusted to 60 mM using KCl (dimer conditions). Elution was detected using UV absorbance, light scattering with a Wyatt Heleos II 18 angle instrument, and finally refractive index using a Wyatt Optilab rEX instrument in a standard SEC-MALS format. Heleos detector 12 was replaced with a Wyatt’s QELS detector for dynamic light scattering measurements. Protein concentration was determined from the excess differential refractive index based on 0.186 RI increment for 1 mg/ml protein solution. Concentrations and observed scattered intensities at each point in the chromatograms were used to calculate the absolute molecular mass from the intercept of the Debye plot, using Zimm’s formalism as implemented in Wyatt’s ASTRA software.

### Steady-state fluorescence spectroscopy

Steady-state fluorescence emission spectra were recorded using a Jasco FP-6500 spectrofluorometer. Samples were measured at 10 μM protein concentration in a 10 mm path-length fluorescence cuvette (Hellma). Fluorescence spectra under monomer and dimer conditions were recorded in 50 mM MOPS buffer, pH 6.4 to 8.2, and in 50 mM MES, pH 5.2 to 7.2, with *I* adjusted to 20 mM using potassium chloride for all solutions. Sample temperature was controlled using a Peltier thermocouple set to 298 K throughout all experiments. Chemical denaturation was performed by manual titration between 0 and 8 M Urea in monomer buffer, i.e. in 50 mM MOPS, pH 7.0, with *I* adjusted to 200 mM using potassium chloride. Salt-dependent measurements were conducted in monomer buffer and *I* adjusted to varying values using potassium chloride.

### Far-UV CD Spectroscopy

Far-UV CD spectroscopy was performed using a Jasco J-815 spectropolarimeter and a 1 mm path-length cuvette (Hellma) containing 10 μM protein. The signal was recorded at 222 nm probing α-helix secondary structure. Chemical denaturation experiments were conducted under the same solution conditions as described above for steady-state fluorescence experiments. Sample temperature was controlled using a Peltier thermocouple set to 298 K. Thermal denaturation was carried out in 50 mM phosphate buffer, pH 7.0, with *I* adjusted to 200 mM using potassium chloride and applying a temperature ramp at a rate of 1 K/min. Salt-dependent thermal denaturation data were acquired using 20 mM phosphate buffer, pH 7.0, with varying *I* adjusted using potassium chloride.

### Time-resolved fluorescence experiments

Association and dissociation kinetics of NTDs were measured using stopped-flow fluorescence spectroscopy. Kinetics of association were measured by recording the native Trp fluorescence signals of NTDs on an Applied Photophysics (SX 18MV) machine equipped with a xenon-lamp as excitation source. Monomeric protein samples were prepared at varying concentrations in 10 mM MES pH 7.0 with *I* adjusted to 150 mM using potassium chloride and rapidly mixed into 20 mM MES buffer, pH 6.0, using a volumetric mixing ratio of 1:11. Each homolog was measured at three different concentrations between 0.1 µM and 1 µM. Each measurement was an average of 20 individual shots. Kinetics of dissociation were measured using chasing experiments following the AttoOxa11 fluorescence of modified NTDs on a SFM-2000 BioLogic stopped-flow machine equipped with a 639 nm diode laser as excitation source. In chasing experiments, 0.1 µM dimeric AttoOxa11-modified NTD sample in 20 mM MES pH 6.0 buffer with *I* adjusted to 60 mM using potassium chloride was chased with 9 µM non-modified NTD prepared in the same buffer. 0.3 mg/mL bovine serum albumin (BSA) and 0.05% Tween-20 were applied as solution additives to suppress glass surface interactions of fluorescently modified protein samples. Six to nine kinetic transients were measured for each homolog and averaged. Kinetics of folding and unfolding of homologs were measured under conditions of chemical denaturation by recording the native Trp fluorescence signals of NTDs using a SFM-2000 BioLogic stopped-flow machine equipped with a 280-nm diode as excitation source. For folding and unfolding kinetics 100 µM protein samples were prepared in buffered solutions containing either zero or six molar urea in 50 mM MOPS, pH 7.0, with *I* adjusted to 200 mM using potassium chloride. Samples were rapidly mixed into urea solutions of varying concentrations applying a volumetric mixing ratio of 1:10 using the stopped-flow machine. Samples were filtered through 0.2 mm syringe filters before measurement. All stopped-flow measurements were recorded at 298 K. Temperature was adjusted using a circulating water bath.

### Data analysis

Equilibrium denaturation data were fitted using the thermodynamic model for a two-state transition between native and denatured states. The spectroscopic signal S can be expressed as a function of denaturant concentration or temperature, here shown as a function of the perturbation *P* ^28^:

1 $$S(P)=\frac{{\alpha }_{N}\,+\,{\beta }_{N}\,\cdot \,P\,+\,({\alpha }_{D}\,+\,{\beta }_{D}\,\cdot P)\cdot \,\exp (-{\rm{\Delta }}{G}_{D-N}(P)/RT)}{1+\exp (-{\rm{\Delta }}{G}_{D-N}(P)/RT)}$$

where α_N_, β_N_, α_D_, and β_D_ are the linearly sloping baselines of native and denatured states, *R* is the gas constant, *T* the temperature, and Δ*G*_D−N_ the difference in free energy between native and denatured state.

In chemical denaturation experiments the folding equilibrium was perturbed by the denaturant urea (*P* = [urea]). ΔG_D−N_ as a function of chemical denaturant is described by the linear-free energy relationship^29^:

2 $${\rm{\Delta }}{G}_{D-N}([urea])={\rm{\Delta }}{G}_{D-N}-{m}_{D-N}[urea]$$

where [urea] is the concentration of urea and *m*_D−N_ is the equilibrium *m*-value that describes the sensitivity of the folding equilibrium to denaturant. Experimental errors of Δ*G*_D – N_ were determined from propagated errors of fitted values of *m*_D−N_ and mid-point concentrations of urea ([urea]_50%_).

In thermal denaturation experiments, the folding equilibrium is perturbed by heat (*P* = *T*) and the free energy of unfolding Δ*G*_D−N_ is described as followed:

3 $${\rm{\Delta }}{G}_{D-N}(T)={\rm{\Delta }}{H}_{m}\cdot (1-\,\frac{T}{{T}_{m}})-{\rm{\Delta }}{C}_{p}[{T}_{m}-T+T\,\mathrm{ln}(\frac{T}{{T}_{m}}\,)]$$

where Δ*H*_m_ is the enthalpy of unfolding at the transition midpoint, T_m_ the mid-point temperature and Δ*C*_p_ is the difference in heat capacity between native and denatured state. For Δ*C*_p_ we applied the empirical value of 14 cal K^−1^ mol^−1^ per residue of a generic polypeptide chain^30^. Changes of stability with varying *I* was estimated using the Schellman formalism assuming conservation of mid-point entropies^31^:

4 $${\rm{\Delta }}{\rm{\Delta }}G=\frac{{\rm{\Delta }}{H}_{m}}{{T}_{m}}\cdot {\rm{\Delta }}{T}_{m}$$

where ∆∆*G* is the change of free energy of unfolding due to the change of *I*, ∆*H* is the enthalpy of unfolding, and ∆*T*_m_ is the difference in the apparent mid-point temperatures of the denaturation curves.

Kinetic transients of folding/unfolding from stopped flow experiments were fitted to a single exponential function containing a linear baseline drift:

5 $$S(t)=a\,\exp (-{k}_{obs}t)+bt+c$$

*S*(*t*) is the fluorescence signal as function of time, *a* is the amplitude and *k*_obs_ the observed rate constant of the transient. The parameters *b* and *c* describe the linear drift of the baseline. *k*_*obs*_ is the sum of the microscopic rate constants for folding and unfolding (*k*_*f*_ and *k*_*u*_). The change of *k*_obs_ as a function of denaturant concentration was analyzed by fitting the data to the chevron model for a barrier-limited two-state transition that follows the linear-free-energy relationship^17^:

6 $$\mathrm{log}\,{k}_{obs}([urea])=\,\mathrm{log}[{k}_{f}\,\exp (-{m}_{TS-D}[urea]/RT)+{k}_{u}\,\exp ({m}_{TS-N}[urea]/RT)]$$

*m*_TS-D_ and *m*_TS-N_ are the kinetic *m*-values of folding and unfolding, respectively, where TS denotes the transition state separating denatured and native free energy wells. *k*_*f*_ and *k*_*u*_ are the microscopic rate constants of folding and unfolding, respectively, under standard solution conditions in the absence of denaturant.

Rate constants of self-association of NTDs were obtained from fitting kinetic transients to a reaction model of protein dimerization^32^:

7 $$\begin{array}{l}2N\,\mathop{\leftrightarrow }\limits^{{k}_{ass}}\,{N}_{2}\\ \frac{d[{N}_{2}]}{dt}={k}_{ass}{[N]}^{2}\end{array}$$

where *N* is the folded, monomeric NTD, *N*_2_ is the dimeric NTD, and *k*_ass_ is the bimolecular rate constant of association. The differential equation can be solved to give^32^:

8 $$S(t)={S}_{t=0}+S\frac{({k}_{app}t)}{(1+{k}_{app}t)}$$

where *S*(t) is the time-dependent signal, *S*_t = 0_ is the signal at time t = 0, *S* is the signal amplitude change, and *k*_app_ is the apparent rate constant. The apparent rate constant is related to the association rate constant *k*_ass_^32^:

9 $${k}_{app}={c}_{N}\cdot {k}_{ass}$$

where c_N_ is the protein concentration in terms of monomer.

Rate constants of dissociation were obtained from fitting kinetic transients of chasing experiments to a mono-exponential rise function.

The equilibrium dissociation constant, *K*_D_, was calculated as:

10 $${K}_{D}=\frac{{k}_{diss}}{{k}_{ass}}$$

### Data availability

The datasets generated and analysed during the current study are available from the corresponding author on reasonable request.
